# CM-Path Molecular Diagnostics Forum—consensus statement on the development and implementation of molecular diagnostic tests in the United Kingdom

**DOI:** 10.1038/s41416-019-0588-1

**Published:** 2019-10-02

**Authors:** Philip S. Macklin, Nischalan Pillay, Jessica L. Lee, Helen Pitman, Sophie Scott, Jayson Wang, Clare Craig, J. Louise Jones, Karin A. Oien, Richard Colling, Sarah E. Coupland, Clare Verrill, on behalf of the CM-Path Molecular Diagnostics working group

**Affiliations:** 1 Department of Cellular Pathology, Oxford University Hospitals NHS Foundation Trust, Oxford, UK; 2 University College London Cancer Institute, London, UK; 3 Strategy and Initiatives, National Cancer Research Institute, London, UK; 4 CM-Path Programme Manager, National Cancer Research Institute, London, UK; 5 Medical Science Liaison (Europe), Guardant Health, London, UK; 6 Molecular Pathology Lead, Department of Cellular Pathology, St George’s University Hospitals NHS Foundation Trust, London, UK; 7 Genomics England, London, UK; 8 Barts Cancer Institute, Queen Mary University of London, London, UK; 9 Department of Pathology, The Queen Elizabeth University Hospital, Glasgow, UK; 10 Nuffield Department of Surgical Sciences, University of Oxford, Oxford, UK; 11 North West Cancer Research Centre, University of Liverpool, Liverpool, UK; 12 National Institute for Health Research (NIHR) Oxford Biomedical Research Centre, Oxford, UK

**Keywords:** Cancer screening, Cancer screening

## Abstract

**Background:**

Pathology has evolved from a purely morphological description of cellular alterations in disease to our current ability to interrogate tissues with multiple ‘omics’ technologies. By utilising these techniques and others, ‘molecular diagnostics’ acts as the cornerstone of precision/personalised medicine by attempting to match the underlying disease mechanisms to the most appropriate targeted therapy.

**Methods:**

Despite the promises of molecular diagnostics, significant barriers have impeded its widespread clinical adoption. Thus, the National Cancer Research Institute (NCRI) Cellular Molecular Pathology (CM-Path) initiative convened a national Molecular Diagnostics Forum to facilitate closer collaboration between clinicians, academia, industry, regulators and other key stakeholders in an attempt to overcome these.

**Results:**

We agreed on a consensus ‘roadmap’ that should be followed during development and implementation of new molecular diagnostic tests. We identified key barriers to efficient implementation and propose possible solutions to these. In addition, we discussed the recent reconfiguration of molecular diagnostic services in NHS England and its likely impacts.

**Conclusions:**

We anticipate that this consensus statement will provide practical advice to those involved in the development of novel molecular diagnostic tests. Although primarily focusing on test adoption within the United Kingdom, we also refer to international guidelines to maximise the applicability of our recommendations.

## Background

Pathology—the study of disease—has evolved significantly since its beginnings with Virchow and a purely morphological description of cellular alterations, to our current ability to make fine-resolution observations at the subcellular/molecular scale.^1^ We can now use this knowledge and modern molecular biological techniques to interrogate human tissue samples in increasingly sophisticated ways, with the ultimate aim of providing more accurate diagnoses that can better guide treatment choices. In the field of cellular pathology, it is now possible to supplement traditional light microscopic assessment of tissue samples with a vast array of information at genomic, epigenomic, transcriptomic, proteomic and metabolomic levels. Thus, molecular diagnostics is now the cornerstone of precision/personalised medicine, in which individual patients receive customised healthcare on the basis of their specific test results, and has the potential to revolutionise patient care and improve outcomes, as exemplified by its use in haematological malignancies.^2^ The application of molecular diagnostics is currently being expanded into other clinical areas; for example, in the United Kingdom (UK), the 100,000 Genomes Project has brought whole-genome sequencing into routine clinical practice by initially applying this technique to cancer and rare diseases.^3^

Despite the promises of molecular diagnostics, significant barriers have impeded its widespread clinical adoption.^4^ Until recently, there has been a lack of national strategy for molecular diagnostic testing with complex commissioning and funding arrangements.^5^ Moreover, the National Health Service (NHS) is currently poorly equipped to embrace fully this healthcare revolution. In particular, the substantial attrition of academic pathology in the UK over the past two decades, coupled with the increasing service demands placed on pathologists, means that many diagnostic laboratories lack the knowledge, expertise and capacity to introduce these new tests efficiently.^6^ In addition, the interaction between clinicians, academia, industry and regulators required to expedite the development of new molecular diagnostic tests and their introduction into clinical practice has not been uniformly present to date.

### Inception of a cross-sector molecular diagnostics forum

In 2016, the National Cancer Research Institute (NCRI) launched its Cellular Molecular Pathology (CM-Path) initiative with the aim of supporting modernisation of pathology in the UK and, in so doing, to help to develop the workforce and infrastructure required to provide nationwide molecular diagnostic services (https://cmpath.ncri.org.uk). To advance pathology in the UK, and thus ensure that patients receive the highest quality of care possible, CM-Path recognises the value of collaborating with industry, regulators and other key stakeholders. To this end, members of CM-Path workstream 4 (‘Technology and Informatics’) convened the first meeting of the CM-Path Molecular Diagnostics Forum on 26th January 2018 at the Royal Society of Medicine in London. The overarching aims of the forum are as follows:

- To define infrastructure, regulatory and workflow requirements for the adoption of molecular diagnostics in NHS pathology laboratories;
- To develop protocols to ensure faster and more efficient implementation of emerging technologies and novel bespoke and validated molecular panels;
- To assist in the education/training of the workforce required to provide high-quality, nationwide molecular diagnostic services;
- To actively engage pathologists with industry and regulators to develop the next phase of molecular diagnostic tests;
- To form links with companies developing software to assist in test interpretation and correlation between molecular findings and clinical outcomes.

Ultimately, we wish to ensure that all patients across the UK have equitable and rapid access to effective molecular diagnostic tests, whether developed by industry or academia. The objectives of this particular meeting, which was attended by 25 individuals including clinicians, academics and representatives from industry and regulatory bodies, were to define a ‘roadmap’ for molecular diagnostic test development and NHS implementation and to identify the challenges (and their possible solutions) that are likely to be encountered during these processes. The meeting commenced with invited case presentations on the development and implementation of new molecular diagnostic tests in rare ophthalmic disease (Professor Graeme Black, University of Manchester) and bladder cancer (Dr Andrew Feber, University College London), providing illuminating ‘real world’ insights into these processes. Summaries of the perspectives of industry and of the National Institute for Health and Care Excellence (NICE) on the current state of affairs were also presented by Jane Coppard (public affairs manager at Roche) and Rebecca Albrow (senior technical adviser in the NICE Diagnostics Assessment Programme), respectively. It was highlighted that NICE diagnostics guidance recommendations are typically made by the Diagnostic Advisory Committee (DAC), an independent decision-making body that bases its recommendations on review of clinical and economic evidence. Once recommendations are made, NICE diagnostics guidance is published on the NICE website^7^ and is disseminated to all stakeholders, which include professional societies, patient organisations and individual clinicians. NICE also creates tools to support the adoption of guidance but there are many factors that can hinder nationwide uptake. Until recently, there has been no systematic method of tracking the use of diagnostics within the NHS, and therefore, the impact of NICE recommendations cannot be directly evaluated.

### Developing a roadmap for the development and implementation of new molecular diagnostic tests

**Fig. 1:**
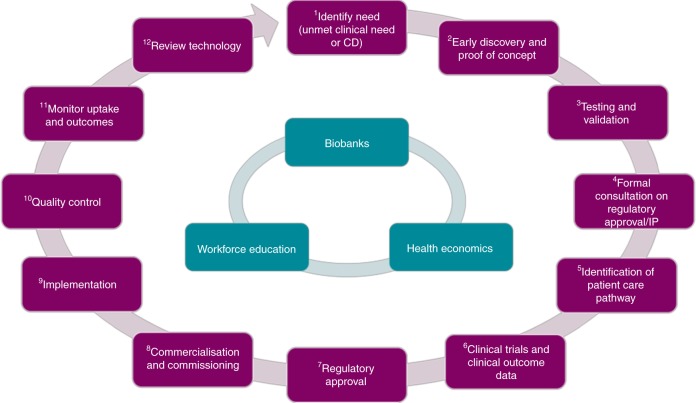
Consensus ‘roadmap’ for the development and implementation of molecular diagnostic tests (key: CD = companion diagnostic*,* IP = intellectual property). (1) Identify need—researchers define a clinical scenario that would benefit from improved diagnostic capabilities or there is a specific need for a companion diagnostic test in parallel to drug development; (2) early discovery and proof of concept— preclinical studies to develop scientific basis of new discovery (we acknowledge that in some cases, this may precede the previous step with clinical relevance only emerging after the initial scientific discovery); (3) testing and validation—further testing, possibly in preparation for human trials (discussed in greater detail by Mattocks and colleagues);^31^ (4) formal consultation on regulatory approval and intellectual property—we recommend discussions with the relevant regulatory bodies and technology transfer offices at an early stage in test development (e.g., the United Kingdom Accreditation Service [UKAS] and the Medicines and Healthcare products Regulatory Agency [MHRA]’s Innovation Office),^32^ to ensure that the correct procedures are being followed and that intellectual property is protected (N.B. must also consider the need for Research Ethics Committee [REC] and Human Tissue Authority [HTA] approval, which is required for testing on human tissue samples); (5) identification of position in patient care pathway—before clinical trials are conducted, it is essential to identify where a new test will fit within the current or redesigned patient care pathway, not just within the United Kingdom but also other countries, especially Europe and the United States of America; (6) clinical trial (conducted according to ethical and regulatory frameworks) and clinical outcome data—a formal clinical trial demonstrating equivalence/superiority to the current ‘gold standard’ diagnostic test may be required; (7) regulatory approval—evidence from proof-of-concept studies and clinical trials will be required to gain relevant regulatory approval (Conformité Européenne marking of In Vitro Diagnostics [CE IVD] in Europe by Notified Bodies and the Food and Drug Administration [FDA] in the United Stated of America); (8) commercialisation and commissioning—after regulatory approval has been granted, the new diagnostic test requires marketing and must be deemed to provide clinical benefit and be cost-effective (i.e. by the National Institute for Health and Care Excellence [NICE]) before it will be commissioned for clinical use within the National Health Service [NHS]; (9) implementation—the new test is implemented in clinical practice; (10) quality control—rigorous quality control and post-marketing surveillance is required to ensure ongoing, high-quality test performance (e.g., in the UK, laboratory accreditation is regulated by UKAS and external assessment is conducted by International Organization for Standardization [ISO] 17043 accredited external quality assurance providers [listed at http://www.eptis.org];^33^ in the specialty of histopathology, this is most commonly undertaken by the United Kingdom National External Quality Assessment Service [UK NEQAS]); (11) monitor uptake and outcomes—it is important to monitor nationwide uptake of new molecular diagnostic tests and to provide firm evidence that the tests provide clinical and/or economic benefit; (12) review technology—ongoing review of the technology, identifying areas for further development/optimisation is essential

In a subsequent breakout session, delegates were grouped by professional background and tasked to create a roadmap describing the stages in the development of a new molecular diagnostic test, from initial concept to clinical implementation. This is particularly important as, compared with therapeutics, the validation and approval processes for diagnostic tests are poorly defined. It quickly became clear that no single group was able to map the entire pathway, immediately justifying the value of arranging this multidisciplinary meeting. Ultimately, a final roadmap was agreed by consensus between the groups (Fig. 1); access to carefully curated tissue specimens through biobanks, health economics and workforce education are key aspects that have central relevance to the entire process. The discussions were very much centred on test development in the UK, although many companies developing such products are multinational or would aim to market them internationally. Although not the focus of the workshop, it was also acknowledged that new diagnostic tests are often introduced alongside new therapies (as ‘companion diagnostics’), so the development of novel molecular diagnostic tests often occurs in parallel to drug development. In this instance, the clinical need would be very clear and specific at the outset but otherwise the overall roadmap would still be similar.

### Challenges to the implementation of new molecular diagnostic tests

The groups were then mixed and asked to identify challenges that are likely to be encountered within the roadmap. Several key themes emerged during this discussion; importantly, a number of innovative solutions were also suggested (Table 1).

**Table 1:** Challenges to the development and implementation of new molecular diagnostic tests and possible solutions to these

Challenge	Solutions
*1. NHS commissioning and standardisation of testing*	
-Limited pathology budgets and current funding structures mean that tests that could improve patient outcomes (and even save money in the long term) may not be funded -Many different tests available (including for the same biomarker), leading to regional variation in testing -The timing of investigations within diagnostic/management pathways can influence the choice of the testing method -When a new therapy has been recommended by NICE, the NHS should commission funding for the companion diagnostic test, but this has not always been the case -Innovative tests that may have a disruptive effect on the local NHS pathways may be less likely to be adopted -Variation in how tissue samples are collected and processed and in how tests are performed and interpreted	-Lobbying for alternative funding sources and changes to how tariffs are allocated -Rigorous assessment of different tests, leading to greater understanding of their strengths and weaknesses, with the aim of uniform adoption of the optimal test -Within reason, flexibility should be encouraged in the new national testing system to ensure that patients have access to the most appropriate test at each point in their care pathway -Ensure that NICE and the NHS are aligned so that when a new therapy is recommended by NICE, there is timely uptake of any companion diagnostic test -Centralised commissioning of testing (as with the recent reconfiguration of genomic testing within the NHS) -Development of SOPs and regular participation in EQA schemes
*2. Ethical and regulatory issues*	
-Requirement for ethical approval and consenting procedures during test development/clinical trials -Uncertainty about the necessary regulatory requirements (e.g. clinical trial authorisation, EU IVDR and US FDA approval) for new molecular diagnostic tests and accreditation of laboratories performing them (uncertainty greater within academia and NHS than within industry) -Uncertainty about how ‘Brexit’ will affect regulation of in vitro diagnostics in the UK (the IVDR, an EU regulation, came into effect in May 2017 and gave manufacturers 5 years to prepare for a new legislation that will require more rigorous assessment of in vitro diagnostic medical devices—it is currently unknown how ‘Brexit’ will affect this)	-Greater clarity with regard to when ethical approval and consent are and are not required (e.g. test development/validation vs. performance assessment of an already-validated test) -Encourage researchers to seek ethical approval at an early stage in test development -Encourage researchers, clinicians and NHS managers to interact with regulators at an early stage in test development and implementation (e.g. through MHRA’s innovation office)^32^ -Promotion of both UK^27^ and European^28^ regulatory guidelines -Lobbying for clarification of legislative/regulatory impact of ‘Brexit’ and possible exemptions from new EU regulations, when appropriate
*3. Information technology*	
-Development of standardised, robust IT infrastructures -Data storage and sharing -Volume and complexity of data	-Investment in IT infrastructure, ensuring new software is compatible with existing ones -Consideration of technical, legal and ethical issues to ensure that data can be safely stored and shared for clinical and research purposes -Development of novel computational approaches (e.g. AI) to facilitate automated analyses
*4. NHS culture*	
-Staff must be aware of emerging technologies and willing to adopt them -Patients should be educated and empowered to ensure that they receive appropriate molecular testing	-Improved nationwide dissemination of information about established/emerging tests and funding sources -Greater communication between specialties (such as at the MDT meeting), to encourage reflex testing by pathologists, when appropriate -RCPath to include NICE recommendations in their best-practice guidelines and datasets -Sharing of case studies demonstrating clinical benefit and cost-effectiveness -A national workshop involving clinical staff, laboratory scientists and NHS managers -Education and mentoring of patients and enhanced communication between patients, clinicians and pathologists (e.g. through the NCRI Consumer Forum)
*5. Education/training*	
-Urgent need to upskill the NHS workforce in molecular diagnostics	-Inclusion of molecular diagnostics in UG medical curricula and increased prominence in PG training, including training of senior staff (CM-Path is actively working to develop training opportunities in molecular pathology)^19,20^ -Cross-discipline and cross-sector training to include clinicians, pathologists, nurses, managers and industry -Identify best-practice examples in molecular diagnostic training from other countries
*6. Monitoring of uptake/response*	
-Lack of systematic monitoring of molecular diagnostic testing in the NHS, leading to a knowledge gap regarding current practices across the UK -Lack of data regarding the clinical impact of test adoption	-NHS genomic reconfiguration to introduce a new molecular diagnostic test directory and commissioning system -Inclusion of molecular diagnostics in quarterly NHS England Innovation Scorecard produced by HSCIC -Mandatory recording of how new tests have influenced patient care (e.g. treatment allocation)

*AI* artificial intelligence, *EQA* external quality assessment, *EU* European Union, *HSCIC* Health and Social Care Information Centre, *IT* information technology, *IVDR* In Vitro Diagnostic Regulation, *MDT* multidisciplinary team, *MHRA* Medicines and Healthcare products Regulatory Agency, *NICE* National Institute for Health and Care Excellence, *NCRI* National Cancer Research Institute, *NHS* National Health Service, *PG* postgraduate, *RCPath* The Royal College of Pathologists, *SOPs* standard operating procedures, *UG* undergraduate, *UK* United Kingdom, *USFDA* United States Food and Drug Administration

A follow-up meeting was held in October 2018 to discuss these challenges in greater detail, and to consider how our roadmap will likely be impacted by the reconfiguration of genomic laboratory services within NHS England that took place that month.^8^ By creating a single national testing network co-ordinated through seven Genomic Laboratory Hubs (GLHs), this reconfiguration aims to expedite widespread adoption of molecular diagnostics into routine clinical practice and to ensure that such tests are conducted to uniform standards, thus providing consistent and equitable care across the country. Building upon the success of the 100,000 Genomes Project, this project forms part of the Government’s Life Sciences Strategy,^9^ and aims to develop a world leading Genomic Medicine Service within the NHS, as well as to support scientific research and innovation more broadly. The new service now includes a National Genomic Test Directory for both cancer and rare and inherited diseases.^10^ This directory specifies which tests are available within the NHS and how they are funded, which patients are eligible to receive these tests and which technology platforms should be used to perform each test. The directory will be updated annually, based on recommendation from a Clinical and Scientific Expert Panel that will evaluate new genomic tests and determine which existing tests should be retired or replaced. The authors believe that this positive development will help with many of the challenges that we have identified but, crucially, it only currently covers genetic testing and not other forms of molecular diagnostics (e.g. infectious disease).

Whilst this new system should help to deliver more uniform nationwide access to molecular diagnostic tests, some scope for local flexibility in testing strategy is likely to be of benefit to patient care. A crucial issue to consider when ordering a molecular diagnostic test is how this test is best integrated into each patient’s individual care pathway and we envisage that local multidisciplinary team (MDT) meetings will continue to play an important role in making such decisions. Some test results are needed more urgently than others and this can influence the type of test selected and whether this is performed locally or sent externally. For example, one-step nucleic acid amplification (OSNA) testing to detect cytokeratin 19 (CK19) mRNA copy numbers in homogenised axillary lymph node samples, as a marker of breast cancer sentinel lymph node metastasis, has been performed in some UK centres for many years, with rapid intraoperative results determining the requirement for nodal clearance as part of a one-step procedure.^11^ Likewise, lung cancer mutation status can have a significant impact upon immediate clinical management and rapid in-house testing can be very useful, particularly in the context of acutely unwell patients or where a prompt initial screening test result can avoid the need to perform further unnecessary tests (e.g. *KRAS* mutations are generally mutually exclusive with *EGFR* and *ALK* mutations in lung cancer, which therefore do not need to be tested for when a *KRAS* mutation is detected).^12^ Initially, MDTs may also wish to arrange local funding for specific tests, rather than incur the time penalty involved in sending samples away. Nevertheless, the majority of molecular diagnostic tests are generally not urgent (e.g screening for Lynch syndrome in colorectal cancer)^13^ and are therefore likely to be best performed in a centralised reference laboratory. Furthermore, over time, we hope that the GLHs will generate evidence to demonstrate that centralised testing can return results in a clinically relevant timeframe for most indications. Another reason to retain local testing might be when a centre has already developed expertise in the performance and interpretation of a specific test, which could not be delivered to the same standard through an associated GLH.

It was felt by forum participants that GLHs could play an important role in the development of novel molecular tests by providing access to high-quality human tissue samples via linked academic biobanks and by assisting in test validation, particularly by facilitating rigorous comparison with established tests and by recruiting patients into clinical trials. Once an evidence base has been established, a key milestone for any new molecular test will be inclusion in the test directory and it is envisaged that this step could be aligned with approval by the NICE DAC. GLHs will also have responsibility for implementing newly approved tests, ideally working in collaboration with each other to ensure optimal quality control, and in monitoring test uptake and downstream clinical effects, for example by transmitting relevant information derived from genomic MDT meetings to a centralised repository of outcome data. Likely future challenges for the GLHs include extending molecular tests to include other ‘omics’ approaches (e.g., epigenomics, transcriptomics, proteomics and metabolomics) whilst at the same time ensuring standardised, high-quality performance of established techniques (e.g. PD-L1 immunohistochemistry in non-small-cell lung cancer, for which several different assays are available).^14^ This may also entail the incorporation of digital pathology, which is currently being promoted via an Innovate UK initiative with the establishment of five centres of excellence for digital pathology, image analysis and artificial intelligence.^15,16^ Such approaches are likely to become part of integrated reporting, bringing together the clinical, morphological, immunohistochemical and molecular data, in order to improve diagnostics and patient management.

Centralised testing offers many benefits but there are also potential downsides to such an approach, and lessons should be learnt from previous reconfigurations of pathology services.^17^ Whilst earlier consolidations have produced cost savings,^18^ a large initial financial investment is often required, for example to cover the cost of new transport networks and to develop the information technology (IT) infrastructure required to connect different hospitals/laboratories. Critically, the NHS workforce remains central to the provision of high-quality diagnostic testing and there is a risk of loss of valuable expertise amongst staff who are not based in GLHs. Furthermore, sending tissue samples away for testing may negatively impact upon the ability of ‘non-hub’ centres to contribute to biobanking activities that are critical to support biomedical research. Given these risks, and to foster a new molecular medicine culture within the NHS, it is imperative that the seven GLHs (and their associated ‘spoke’ hospitals) adopt a collaborative, rather than competitive, approach to service delivery. Importantly, shared leadership by pathology, genetic and clinical teams will be needed to deliver a truly integrated service.

Nationwide delivery of a ‘cutting-edge’ molecular diagnostic service will require large-scale upskilling of the current laboratory workforce, as well as amendments to the training of medical students, junior doctors and clinical scientists. With this requirement in mind, CM-Path, in collaboration with other relevant organisations, is actively working to develop training opportunities in molecular pathology.^19,20^ Importantly, a requirement for formal molecular pathology teaching is now included in the Royal College of Pathologists (RCPath) ‘Curriculum for Specialty Training in Histopathology’;^21^ a 2-week molecular pathology attachment for histopathology trainees is now advocated^22^ and trainee knowledge of this area will be evaluated both through workplace-based assessment and formal professional examinations. The curriculum is currently undergoing further revision and it is envisaged that molecular pathology will feature even more prominently in the next iteration. In parallel, Health Education England (HEE), in partnership with several leading UK universities, provides formal postgraduate qualifications in genomic medicine as part of its Genomics Education Programme, as well as numerous other online-learning resources (https://www.genomicseducation.hee.nhs.uk). In addition, a range of professional training courses in molecular pathology are also available: ‘*Molecular Pathology and Diagnosis of Cancer’* delivered by the Wellcome Genome Campus and RCPath,^23^ ‘*UK Molecular Diagnostics Training School*’ delivered by the Nottingham Molecular Pathology Node,^24^ ‘*Molecular Pathology Study Day*’ organised by the British Division of the International Academy of Pathology (BDIAP)^25^ and ‘*Getting to Grips with Genomics*’ which is a joint initiative between CM-Path, RCPath and HEE, and importantly, provides education in molecular pathology to both trainees and trainers alike.^26^

Finally, legal, accreditation and regulatory frameworks must be considered when selecting or developing new molecular diagnostic tests. New in vitro diagnostic devices (IVD) must be approved before clinical adoption; regulatory guidelines for such approval exist both within the UK^27^ and the European Union (EU).^28^ In the UK, the Medicines and Healthcare products Regulatory Agency (MHRA) is responsible for ensuring that medical devices are safe for clinical use. Currently, there is a Europe-wide transition to the new EU Regulation on In Vitro Diagnostic Medical Devices 2017/746.^29^ This regulation sets out a new pathway for certification that will be carried out by approved notified bodies and Conformité Européenne In Vitro Diagnostic (CE IVD) approval is a sign of conformity with European standards. Whilst still to be confirmed, it is likely that these changes will apply in the UK even after its withdrawal from the EU. In the UK, all molecular assays and laboratory processes must also be accredited by the United Kingdom Accreditation Service (UKAS) through meeting a range of different International Organization for Standardization (ISO) requirements. UKAS also requires that IVDs undergo external quality assessment (EQA), with such quality control exercises most commonly conducted by the United Kingdom National External Quality Assessment Service (UK NEQAS). In the United States of America, IVDs are classified based on likely patient risk and are usually required to undergo premarket approval (PMA), unless there is a specific exemption.^30^ Through the Molecular Diagnostics Forum, for example, CM-Path is working closely with the MHRA and The British In Vitro Diagnostic Association (BIVDA) in order to ensure that regulators are involved at an early stage in the development of new diagnostic tests.

### Conclusions and future perspectives

Our NCRI CM-Path Molecular Diagnostics Forum meetings proved to be highly constructive in identifying strengths and weaknesses in the application of molecular pathology across the NHS and the group is committed to facilitate continued collaboration between pathology (in both the NHS and academia), industry and regulators. To our knowledge, this is the first cross-sector attempt at defining the roadmap for molecular diagnostic tests, from conception through to deployment and use in accredited laboratories within the NHS. Whilst this process is currently complex, we believe that many of the challenges that we have identified can be overcome through closer collaboration between key stakeholders and with the network of GLHs. The next forum meeting will have a specific emphasis on addressing optimal sample handling for molecular testing, how the new ‘hub and spoke’ arrangement of GLHs will impact upon specimen journey from patient to laboratory and how molecular testing at GLHs can be potentially integrated with digital pathology being performed at the above-mentioned five new centres. Lessons learned will be integrated into the roadmap, further developing molecular diagnostic capabilities in the UK.

CM-Path would be delighted to hear from any individual or group who feel that the Molecular Diagnostics Forum is relevant to their work and who would like to attend future meetings—please email cmpath@ncri.org.uk to get in touch.

## Data Availability

Not applicable
